# Low-cost 3D printed lenses for brightfield and fluorescence microscopy

**DOI:** 10.1364/BOE.514653

**Published:** 2024-03-07

**Authors:** Jay Christopher, Liam M. Rooney, Mark Donnachie, Deepak Uttamchandani, Gail McConnell, Ralf Bauer

**Affiliations:** 1 Department of Electronic and Electrical Engineering, University of Strathclyde, Glasgow, UK; 2 Strathclyde Institute of Pharmacy and Biomedical Sciences, University of Strathclyde, Glasgow, UK

## Abstract

We present the fabrication and implementation of low-cost optical quality 3D printed lenses, and their application as microscope objectives with different prescriptions. The imaging performance of the 3D printed lenses was benchmarked against commercially available optics including a 20 mm focal length 12.7 mm diameter NBK-7 plano-convex lens used as a low magnification objective, and a separate high magnification objective featuring three 6 mm diameter NBK-7 lenses with different positive and negative focal lengths. We describe the design and manufacturing processes to produce high-quality 3D printed lenses. We tested their surface quality using a stylus profilometer, showing that they conform to that of commercial glass counterpart lenses. The 3D printed lenses were used as microscope objectives in both brightfield and epi-fluorescence imaging of specimens including onion, cyanobacteria, and variegated *Hosta* leaves, demonstrating a sub-cellular resolution performance obtained with low-cost 3D printed optical elements within brightfield and fluorescence microscopy.

## 1. Introduction

Biological imaging research is fundamental in advancing both science and healthcare. To make the resulting developments more widely available both low-cost and open-source microscopy developments can help with circumventing the high economic constraints of conventional commercial biomedical research systems [1]. For some biomedical imaging systems, costs are driven higher due to the requirement for specific optical elements, which can often come at a premium due to their bespoke manufacturing processes and custom design requirements. These factors impose a barrier to entry that constrains biological and diagnostic imaging in low-resource settings. Glass lens manufacturing processes traditionally require grinding and polishing steps, which are time-consuming and costly [2–4]. Alternatively, injection molded lenses can minimize these costs through mass-scale lens production, however high precision molds must initially be made before high-quality optics can be created, which itself can be both expensive and time-consuming [5–7]. Additionally, when considering the developments of prototype, non-standard and free-form lens geometries employed within imaging research [8–10] in conjunction with the relatively limited customer-base per unique lens specification, the costs in manufacturing each lens or lens mold increases further still. These costs are then passed onto the consumer, slowing, or stopping the participation of biomedical researchers with minimal resources.

Recently, additive manufacturing has demonstrated the potential of 3D printing optical quality free-form lens geometries [11]. Many of the 3D printed optics approaches developed thus far are based on two-photon polymerization (TPP) techniques, such as direct laser writing (DLW), due to their exceptionally high printing resolution and minimal requirements for post-processing [12–14]. However, although TPP can manufacture parts with sub-micron detail, the economic cost per part is significantly higher than other 3D printing methods because expensive pulsed laser sources are necessary for the fabrication process. Additionally, the large spatial footprint and time per print from TPP systems is often a constraint whilst allowing only millimeter-scale parts to be manufactured, albeit with extremely fine detail [12]. Though TPP is an impressive manufacturing technique, it still only caters toward higher-budget research, especially for optical quality at the macro-scale.

Stereolithography (SLA) is a low-cost 3D printing technique capable of manufacturing optical components using ultraviolet-excited photopolymerizing resins in a layer-by-layer process [15,16,17]. This stepwise process can require additional post-processing to obtain optical clarity through post-processing approaches like spin-coating or dip-coating with liquid resin, or subtractive methods such as polishing the lens surface with fine grit paper [18,19]. Custom techniques have been developed previously to improve the capabilities of low-cost optical printing, such as iterative learning printing algorithms which adjust grayscale pixel values throughout the print, or image pattern defocusing to smooth the lateral pixel gaps, both of which minimize the occurring staircase effect [20,21], yet still include a coating post-process step. Aside from coating techniques, liquid immersion lens molding can produce lens geometries theoretically invariant of diameter or complexity and without the need for post-processing [22,23]. These low-cost manufacturing methods provide the opportunity to repeatably manufacture optical components with tailored uses within microscopy setups.

Individual lenses or lenslet arrays, manufactured using SLA techniques, have been applied so far to image chrome lithography resolution targets [15,16,24] or by analyzing the shape of a transmitted laser beam [21]. Although these tests are integral for quality benchmarks of in-house lens fabrication, the application and full performance evaluation of 3D printed lenses in biological or biomedical imaging has so far been neglected. We therefore present the potential of low-cost LCD desktop 3D printing for optical quality lens manufacturing when used in biological applications and emphasize their use within fluorescence microscopy which is commonly used for biological imaging. From these results, numerous other applications for the presented 3D printed elements are directly possible, such as beam shaping applications. We demonstrate the use of both single and 4-lens microscopy objectives using affordably produced lenses of varying geometries across both brightfield and fluorescence imaging. The imaging performance of each lens geometry was evaluated in brightfield illumination using a 1951 USAF target for resolution and contrast evaluation, as well as cyanobacteria, variegated *Hosta Undulata* leaf, and iodine-stained onion cell samples. We also show fluorescence epi-illumination and imaging using the single lens and 4-lens 3D printed microscopy objectives to demonstrate the potential of 3D printed lenses in biological fluorescence imaging.

## 2. Materials and methods

### 2.1 3D printing and post-processing protocol

3D printed lens geometries were created in a mechanical CAD software (Autodesk Inventor Professional 2023), with identical geometry to off-the-shelf components for cross-comparison. The design files were translated to printer-readable file formats using a free slicer (Chitubox basic) and printed using an Elegoo Mars 2 consumer grade 3D printer with 10 µm layer step size and lateral pixel size of 50 µm. Clear Resin (RS-F2-GPCL-04, Formlabs) was used as material, with print settings optimized for the material choice and resin material properties having been described previously [25].

**Fig. 1.**
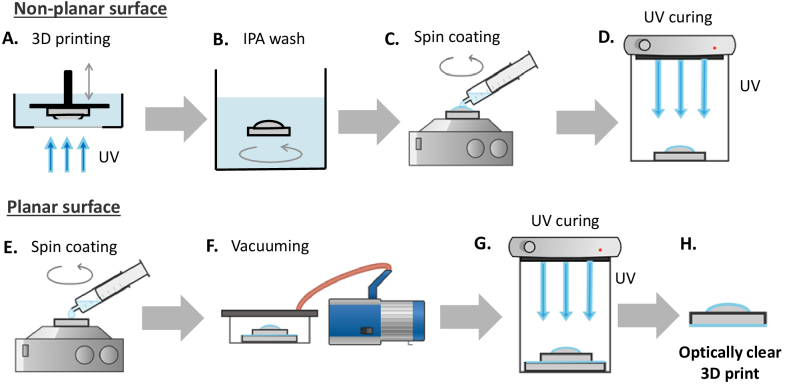
Schematic of 3D printed optics manufacturing process. (A)-(D) Manufacturing an optically clear non-planar surface; (E)-(G) Manufacturing an optically clear planar surface; (A) 3D printing schematic showing the layer-by-layer technique using incident UV light; (B) Cleaning stage using isopropyl alcohol (IPA) to remove residual resin; (C) spin coating non-planar surface by pipetting resin onto surface prior to spinning; (D) non-planar spin coated surface cured with UV light; (E) resin pipetted onto glass slide for spin coating; (F) resin-coated glass slide and 3D printed planar surface placed in contact and vacuumed together; (G) UV curing of resin coated slide and planar 3D printed surface; (H) schematic of resultant optically clear 3D printed part, shown here as a plano-convex lens.

The fabrication process to obtain optical quality non-planar components is shown in Fig. 1(A)-(D). The completed print was cleaned with 100% isopropyl alcohol (IPA) and blow-dried with compressed air before the curved side was spin-coated (Ossila L2001A3-E463-UK spin coater) with a secondary clear resin (UV resin crystal clear, Vida Rosa) with faster cure times. Coating parameters such as spin speed and spin time are intrinsically related to the printed dimensions, such as the surface area and radius of curvature, and were therefore independently optimized for each lens (see Table 1, where the printed version of a Thorlabs lens was suffixed with ‘-P’). To ensure processing optimization, a measurement of the radius of curvature is essential in iteratively and quantitatively tuning a lens of any diameter *d* to its optimal spin-speed and time. UV curing for 12 minutes was performed using 405 nm and 385 nm LED excitation (Elegoo Mercury Plus 2-in-1 wash and cure station). After curing the curved surface, the lens was again washed with IPA and blow-dried with compressed air.

**Table 1.** Spin-coating characteristics for each 3D printed lens.

Lens Name	#1, LA1074-P	#3, LA1116-P	#4, LC2969-P	#5, LA1222-P
**Coating Speed (RPM)**	1800	2600	4000	2800
**Coating Time (s)**	10	14	10	15
**Coating Amount (mg)**	120	4	4	4

The planar surface coating (Fig. 1(E)-(H)) is created by spin-coating a glass microscope slide with liquid resin (RS-F2-GPCL-04, Formlabs) at 1400 RPM for 10 seconds, and then placing the cleaned planar surface onto the slide to enable best planar surface quality [15].To remove macroscopic air bubbles formed within the resin between the printed lens and glass slide, the combination was left in a vacuum chamber of –0.9 bars for 30 minutes, or until all bubbles had been removed. The lens-slide combination was then UV cured for 8 minutes using the same Mercury Plus wash and cure station as before, and the finished lens was removed from the glass slide using a freezer spray to leverage differential thermal expansion between the printed plastic and glass slide. Some manual leveraging with a scalpel at the 3D printed lens’ edge was additionally required at times. To manufacture and process the described lenses, the material costs were found to be under $ 0.18 per lens.

To ensure processing optimization, the use of a contact stylus profiler, optical surface profiler, or any similar method of determining the radius of curvature of the manufactured non-planar surface is essential as it allows quantitatively tuning a lens of any diameter *d* to its optimal spin-speed and time. Lenses of 12.7 mm diameter and 20 mm designed focal length were used to iteratively evaluate the relationship between spin-speed and obtained radius of curvature, where it was found that larger radii of curvature generally required lower spins-speeds than smaller radii of curvature.

### 2.2 Evaluation of surface quality using a stylus contact approach

The optimal spin speed and spin time for the post-process lens coatings were evaluated based on a parametric sweep using a Tencor Alpha Step IQ Stylus profiler with a 5 µm tip to characterize the surface profile of each lens, and a MATLAB script was written to determine any radius of curvature mismatch between the 3D printed lenses and their commercial counterparts. A limit of the contact approach, however, is its relatively short mm-scale lateral measurement distances due to the limited vertical measurement range while maintaining a high accuracy axial measurement resolution.

### 2.3 3D printed single and 4-lens microscope objective design and evaluation

**Fig. 2.**
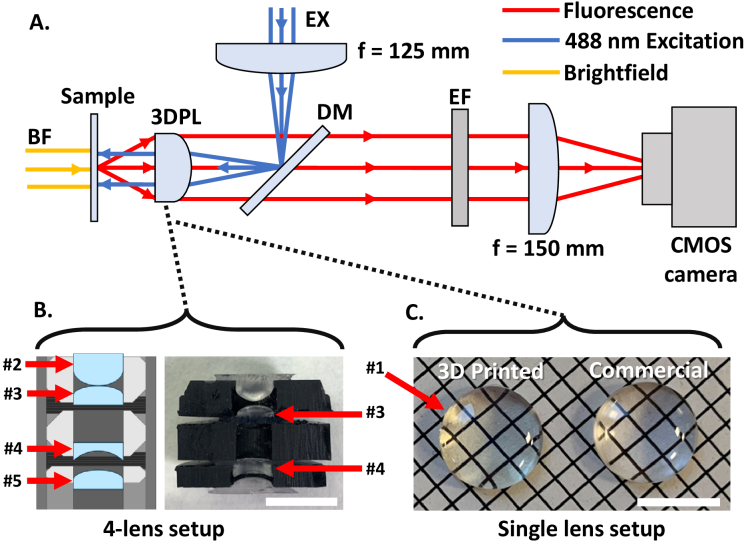
Microscope setup for brightfield and fluorescence image comparison of 3D printed and commercial single and 4-lens objectives. (A) Schematic of the microscope setup using either a group of 4 singlet lenses or a single plano-convex lens as the primary objective, shown as (B) and (C) respectively. BF –collimated white light source for brightfield illumination; EX – 488 nm collimated multi-mode laser input; 3DPL – 3D Printed objectives under test; DM – dichroic mirror; EF – emission filter. (B) Schematic of 4 singlet lenses and photograph to match schematic. #2 Commercial aspherical f =  + 6 mm lens; #3 f =  + 10 mm plano-convex lens; #4 f = -6 mm plano-concave lens; #5 f =  + 15 mm plano-convex lens. (C) Photograph of 12.7 mm diameter, 3D printed f =  + 20 mm plano-convex lens and commercial counterpart with identical specifications. Numbers #1, #3 and #4 show which lenses are used to test 3D printed lenses in imaging. Scale bars = 10 mm.

A simple custom microscope setup with fluorescence epi-illumination and brightfield Köhler illumination was used as a testbed for evaluating the imaging performance of the 3D printed optical elements in microscopy applications (Fig. 2). The testbed consisted of a 30 mm optical cage including a 150 mm achromat tube lens (Thorlabs AC254-150-A) and an industrial CMOS camera (IDS U3-3060CP) to capture the test images, with an empty cage plate near the sample to integrate either commercial or 3D printed objectives. Köhler illumination was created through a separate cage system using a white light LED source (Lumiled Luxeon C Starboard) and multiple lenses and apertures to produce uniform illumination with variable numerical aperture. Epi-fluorescence illumination was provided by a multi-mode laser diode module of 488 nm wavelength (Odicforce OFL-488) which was fibre coupled through a 5 m long multimode fibre (Thorlabs M43L05). The output light from the fibre was focused into the test samples using a commercial f =  + 125 mm achromatic lens (Thorlabs AC254-125-A) and the 3D printed microscope objective under test. For fluorescence imaging, a dichroic mirror (Chroma ZT405/488/561/640rpcv2) was used with two 500 nm long pass emission filters (Thorlabs FELH0500) placed in the infinity space before the tube lens. The 3D printed objectives were placed to create a telecentric imaging setup.

**Fig. 3.**
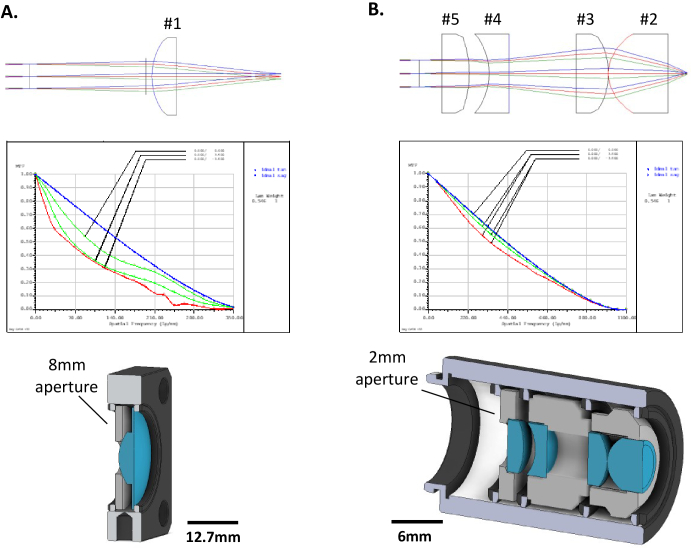
Raytrace diagrams, modulation transfer function (MTF) and 3D assembly schematics for both single lens and 4-lens objective. (A) Single lens objective with entrance aperture of 8 mm diameter at 1 mm distance from the plano-convex lens; the MTF shows clear image deterioration due to the basic spherical lens design; the 3D assembly shows the 3D printed support to house the lens in a 25.4 mm cage plate. (B) 4-lens objective with two plano-convex, one plano-concave and one aspheric lens and entrance aperture of 2 mm diameter at 1 mm distance from the proximal end lens; the MTF shows an almost diffraction limited performance; the 3D assembly highlights the multiple spacers for axial alignment of the lenses.

The two types of primary microscope objectives using 3D printed elements were designed using the raytracing software Optalix to determine optimal lens placements and configurations. A single plano-convex f =  + 20 mm lens (Thorlabs LA1074), here-in called ‘lens #1’, was used as single element objective (see Fig. 2(C)) for a comparison of the 3D printed and commercial glass lens. A 4-lens objective geometry was additionally designed based on 6 mm diameter singlet element lenses (see Fig. 2(B)). The objective consisted of an f =  + 6 mm aspheric front lens (#2, Thorlabs APL0606), a f =  + 10 mm plano-convex secondary lens (#3, Thorlabs LA1116) which is referred to as ‘lens #3’ within this work, a f = -6 mm plano-concave compensation lens (#4, Thorlabs LC2969) called ‘lens #4’ from this point, and a final f =  + 15 mm plano-convex lens (#5, Thorlabs LA1222). It should be noted that only lenses #3 and #4 replace their commercial counterparts within the 4-lens objective, with the other two lenses remaining commercial in the presented iterations of the objective. Element spacings were optimized using the Raytracing software Optalix (see Fig. 3), with a theoretical minimum optics limited imaging resolution of around 1 µm. For the 3D printed single lens objective using lens #1 the theoretical NA is 0.1 while for the 3D printed 4-lens objective it is 0.29. For the commercial 4-lens objective the theoretical NA is slightly higher at 0.35 due to the commercial lens #4 having a different refractive index to the 3D printed version (N-SF11 refractive index of 1.78, compared to Formlabs Clear refractive index of 1.51) due to the limited availability of off-the-shelf 6 mm diameter plano-concave lenses with the right prescription. The stock lens geometry designs for each lens were taken from the catalogue on the Thorlabs website and used as STL files for printing.

Both the 3D printed and commercial lens #1 objectives were housed in a 30 mm cage plate (see Fig. 3(A)) fixed in a telecentric arrangement in the imaging system, with an 8 mm diameter aperture placed on their convex face to act as an aperture stop to achieve optimal resolution conditions. The commercial and 3D printed lenses within the 4-lens objectives were housed in a 12.7 mm lens tube with custom 3D printed spacers (see Fig. 3(B)), printed at a 50 µm resolution, with chamfered edges and retaining rings which all help to minimize lateral offsets between the lenses and ensures the distance between each lens matched closely the objective design parameters. The minimization of lateral offset errors was essential as the ray trace simulations indicated that displacements >50 µm in lateral directions significantly reduced the achievable image quality. A 2 mm diameter stop aperture was integrated into the 3D printed lens holder design and positioned 1 mm after the final 6 mm lens (Fig. 3(B)) to avoid overfilling, improving resolution and contrast.

### 2.4 Sample preparation

For brightfield imaging a 1951 USAF resolution target was used (Thorlabs R3L1S4P) along with a sample of variegated *Hosta* (*Hosta Undulata*), a freshwater cyanobacteria sample, and an onion cell sample. For the variegated *Hosta* sample the leaf cuticle was removed with a scalpel. This sample was then rinsed with deionized water to remove any debris that occurred during membrane removal. A small section (5 mm by 5 mm) was placed onto a #1.5 coverslip with a small amount of deionized water and sealed with a glass microscope slide. To mount the cyanobacteria, the specimen was washed with deionized water to remove detritus and the cyanobacteria were spread using tweezers on a #1.5 coverslip before being sealed onto a glass microscope slide using nail varnish. The onion cells were prepared by dissecting a 5 mm x 5 mm section of the abaxial epidermis from a brown onion and mounting the membrane (5 mm x 5 mm) onto a #1.5 coverslip with 100 µl of neat Lugol’s iodine solution (62650; Merck, Germany) to produce a stained specimen.

For fluorescence imaging the same *Hosta* and cyanobacteria samples were imaged, making use of the autofluorescence of chlorophyll in the chloroplasts for contrast.

For point spread function evaluation a sample of 500 nm microbeads (Thermo Scientific Fluoro-Max green G500) was used. Five microliters of the aqueous bead solution was placed on a #1.5 coverslip before being sealed with nail varnish on a microscope slide and imaged.

## 3. Results

### 3.1 3D printed lens characteristics

**Fig. 4.**
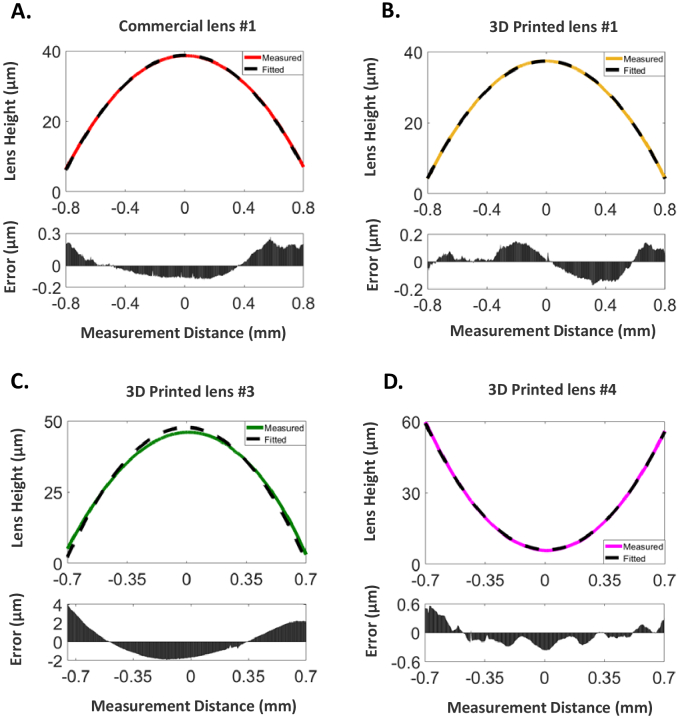
Geometrical characteristics of the 3D printed lenses. (A) Surface profile of the commercial version of lens #1; (B) Surface profile of the 3D printed version of lens #1; (C) Surface profile of the 3D printed lens #3; (D) Surface profile of the 3D Printed lens #4

The surface profiles of the 3D printed lenses for the single and 4-lens microscope objective design were measured using the stylus profiler within a cleanroom environment, with the profile of the commercial lens #1 glass polished lens also measured as comparison (Fig. 4(A)). For lens #1, the resulting measured radii of curvature are 9.67 mm for the 3D printed lens and 9.94 mm for the commercial glass lens. The shape error relative to an ideal spherical shape over a 1.6 mm central diameter is maximally 270 nm and 152 nm respectively, with surface roughness below 70 nm RMS for both lenses.

For the 3D printed lenses #3 and #4 within the 4-lens objective, the measured surface shapes are shown in Fig. 4(C) and (D). The shape error for lens #3 shows the highest maximal error of 4.05 µm which could be due to the slower spin speeds required for this lens in comparison to lens #4 which was spun at nearly double the rotational speed and has a maximum error of 600 nm. For all three cases, the surface roughness is below 150 nm RMS. The measured radii of curvature are for all three cases within 94% of the design specifications based on the original Thorlabs design files, with at least three replicates having been measured for each lens type. For identical non-planar surfaces, the spin-coating speed both at its optimized and non-optimized point exhibited a surface roughness <120 nm. The flat sides of all lenses have additionally a surface roughness <50 nm [26]. Additionally, the refractive index of Formlabs resin is 1.51 at 515 nm excitation [25] with Vida Rosa having shown negligible difference [17]. This should offer only minimal aberration contributions. Formlabs Clear resin does exhibit some autofluorescence behavior, which was found to be much higher for shorter wavelengths of light in the visible. This is unsurprising as the resin is cured at near UV wavelengths. For an excitation wavelength of 525 nm, the recorded autofluorescence is significantly less intense in comparison to an excitation wavelength of 455 nm, approximately by a factor of 30. When imaging a fluorescent sample, the difference in imaging with these wavelengths would be an overall increase in background relative to the sample.

### 3.2 Brightfield imaging with 3D printed lenses

**Fig. 5.**
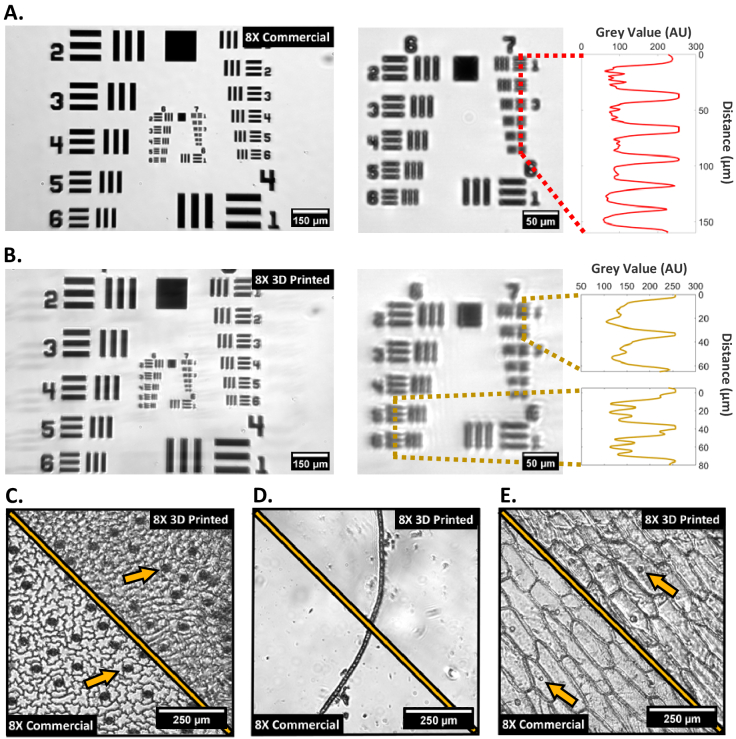
Brightfield imaging using lens #1 as an 8X objective. (A) left - Full FOV 1951 USAF target using commercial lens #1 as objective; middle – central cropped ROI from commercial lens image on left; right – line graph taken through group 7 elements 1-6; (B) left – Full FOV 1951 USAF target using 3D printed lens #1 as objective; middle - central cropped ROI from 3D printed lens image on left; right - line graph taken through group 6 elements 5-6 and group 7 elements 1-2; (C-E) plant cell images using the commercial or 3D printed 8X configuration of: variegated *Hosta* (C), cyanobacteria filament (D) and iodine stained onion cells (E).

Using the microscope setup shown in Fig. 2(A) with the Köhler brightfield transmission illumination and with the lens #1 acting as the objective leads to an approximately 8X magnification. The resolution test target shows comparable contrast between the commercial and 3D printed lens (Fig. 5(A) and (B) respectively) over their 1.4 mm by 0.9 mm field of views. For the 3D printed implementation, a small reflection shadow is visible which is attributed to diffraction effects due to voxel patterns within the 3D printed optics. A small degradation in the image quality is visible, with a zoom-in on groups 6 and 7 of the resolution target, showing that for the 3D printed lens implementation group 6-6 can be clearly identified, leading to a resolution of around 6 µm, while the commercial lens implementation can resolve down to group 7-1, leading to a resolution of around 4.5 µm. In the digitally zoomed region of interest, the commercial line profile shows slightly higher contrast compared to the 3D printed lens due to slight blurring. Brightfield transmission images of variegated *Hosta*, filamentous cyanobacteria, and onion skin cells stained with iodine (see Fig. 5(C)-(E)) show clearly resolvable stomata (see arrow in Fig. 5(C)) and cell structure for the first case, individual compartments in the cyanobacteria filament, and clearly defined cell walls and nuclei (see arrow in Fig. 5(E)) in the onion cells for both commercial and 3D printed imaging configurations. The slightly reduced resolution of the 3D printed lens shows extra blurring around the cell walls in the variegated *Hosta*, cyanobacteria and onion compared to the commercial lens images.

**Fig. 6.**
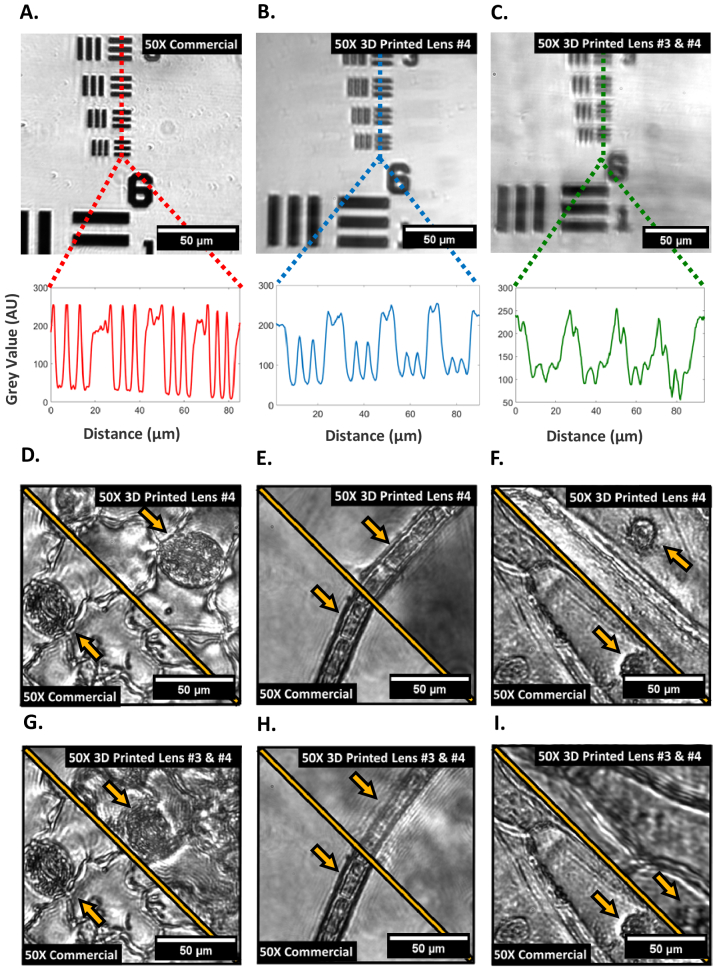
Brightfield images using the 50X 4-lens objective. (A-C) 1951 USAF Group 7 target images with line plot profiles underneath using commercial 4-lens objective configuration. (A) Fully commercial 4-lens objective; (B) lens #4 replaced with a 3D printed version; (C) lenses #3 and #4 replaced with 3D printed versions; (D-F) plant cell image comparisons between the commercial 50X configuration and the 50X configuration using a 3D printed version of lens #4 of: variegated *Hosta* stomata (D), cyanobacteria (E) and iodine stained onion (F); (G-I) plant cell image comparisons between the commercial 50X configuration and the 50X configuration using 3D printed versions of lenses #3 and #4 of: variegated *Hosta* stomata (G), cyanobacteria (H) and iodine stained onion (I).

Using the same microscope setup as shown in Fig. 2(A), but with the 4-lens objective configuration shown in Fig. 3(B) instead of lens #1, 3D printed lenses #3 and #4 were used to sequentially replace one or more commercial glass elements to evaluate the impact of using multiple 3D printed elements in succession in a custom microscope objective. Their performance is compared against a completely commercial lens element counterpart using the 1951 USAF target to examine resolution and contrast as shown in Fig. 6(A)-(C). The magnification for the all-commercial implementation is approximately 47X, which is kept consistent when replacing lens #4 (Fig. 2(B)) with a 3D printed element, however replacing both lens #3 and lens #4 with 3D printed elements increases the magnification to 50X. The shift in magnification is likely a result of the variation in refractive index of the commercial #4 lens vs the 3D printed #4 lens (1.78 vs 1.51) combined with sub-millimeter-scale axial displacements of single or multiple lenses in the 3D printed 4-lens setup which, at the high magnification, compounds into the shown differences in magnification. Additionally, diffraction effects are again evident in the 3D printed lens images, shown by faint replicate areas of the target laterally displaced across the FOV. Overall, the use of one or more 3D printed lens elements at the higher magnification provides resolution of the smallest line-pairs on the resolution target, indicating an imaging resolution of <2 µm which allows resolving of sub-cellular details using 3D printed optical elements. With a higher number of 3D printed lenses used in the 4-lens objective, an increased blurring and reduction in resolution is however visible, originating from manufacturing tolerances and potential minor internal scattering within the volume of the 3D printed elements, though these issues were expected from the low-cost manufacturing method.

Figure 6(D)-(I) shows the application of the 4-lens custom objectives with the same biological samples as previously. In these cases, the images of the single and 4-lens 3D printed versions have been scaled to remove the magnification mismatch and allow direct comparison of contrast and image quality. Figure 6(D)-(F) has a direct comparison between an all-commercial implementation and one that replaces a single lens element with a 3D printed version. For all three imaging examples similar contrast can be seen, with minor extra shadowing around the cell membranes due to the mentioned diffraction effects of the 3D printed lens. In all cases, cell nuclei e.g., in the guard cells of the *Hosta* stomata, and membranes (see Fig. 6(D)-(F) arrows) are clearly visible and individual compartments of cyanobacteria filaments are distinguishable, including their internal morphology. When adding a second 3D printed lens (Fig. 6(G)-(I)) the imaging results show further blurring and potential multiple diffractive effects, which are specifically visible for the single cyanobacteria filament. The contrast and resolution degradation are leading to a reduced visibility of the internal nuclei structure in *Hosta* guard cells and the onion cells, while the main details are still resolvable.

### 3.3 Fluorescence imaging with 3D printed optics

**Fig. 7.**
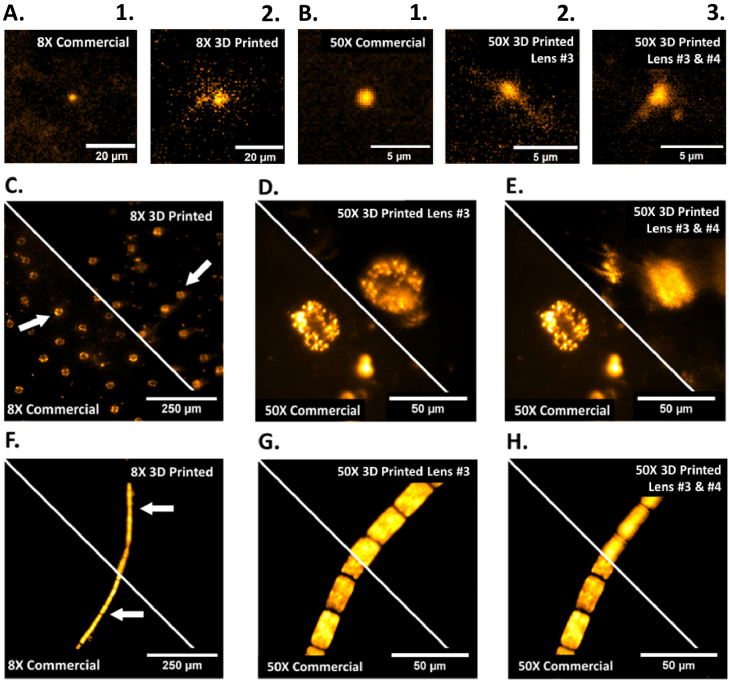
Fluorescence images taken using commercial and 3D printed lens combinations. (A) 500 nm fluorescent beads imaged with the 8X objective. (1) Commercial 8X, (2) 3D printed 8X; (B) 500 nm fluorescent beads imaged with the 50X objective combinations. (1) Commercial 50X, (2) 50X using 3D printed lens #4, (3) 50X using 3D printed lenses #3 and #4 ; (C-E) Variegated *Hosta* stomata auto-fluorescence under 488 nm excitation; (C) commercial vs 3D printed 8X; (D) commercial 50X vs 3D printed lens #4 within 50X ; (E) commercial 50X vs 3D printed lenses #3 and #4 within 50X ; (F-H) cyanobacteria auto-fluorescence under 488 nm excitation; (F) commercial vs 3D printed 8X; (G) commercial vs 3D printed lens #4 within 50X ; (H) commercial vs 3D printed lenses #3 and #4 within 50X.

To show for the first time the performance of using 3D printed lens elements in an epi-fluorescence microscope system, images were captured using a 488 nm multi-mode laser excitation with laser illumination and fluorescence collection through the 3D printed elements. A laser excitation power of 500 µW was measured at the sample for the 8X magnification commercial and 3D printed lens #1 objectives, and a maximum excitation power of 5 mW for the approximately 50X magnification objectives with 3D printed lens elements #3 and #4. The increased power was necessary to compensate for consistency in the signal to noise ratio between the captured fluorescence using lens #1 and the 4-lens fluorescence. Each of the fluorescence images were captured without camera binning at 100 ms exposure time. Using 500 nm sub-resolution fluorescence microbeads, a point spread function (PSF) comparison of the 8X commercial and 3D printed objective configuration is shown in Fig. 7(A), together with the PSFs for three 4-lens 50X objective configurations (all commercial, 3D printed lens #4, and 3D printed lenses #3 and #4) in Fig. 7(B). Significantly degraded PSFs can be seen using the 3D printed lenses as a result of aberrations and scattering effects. The measured fluorescence resolution concurred with Optalix simulations of the PSFs, with the resolution being 3.0 µm and 5.5 µm for the commercial and 3D printed lens #1 objectives, and 0.93 µm, 1.1 µm and 1.4 µm for the commercial, 3D printed lens #4 and 3D printed lens #3 and #4 4-lens objectives. To show the biologically relevant application of the two different magnification objectives, samples of variegated *Hosta* and cyanobacteria filament were imaged using the autofluorescence of chlorophyll as the contrast agent (see Fig. 7(C)-(H)). Comparing the commercial lens and 3D printed lens for the 8X configuration (Fig. 7(C) and (F)) shows good image contrast and the *Hosta* stomata remain easily resolved as can be seen from their characteristic shape and distinct chloroplasts. Additionally, the cyanobacteria has individual cells containing chloroplasts easily discernable from one another in both the commercial and 3D printed cases, though some blurring is observed in the 3D printed version along the edges of the cyanobacteria filament. For the 50X 4-lens objective both the commercial version and the 3D printed version using printed lens #4 allow the entire *Hosta* stomata to be resolved (Fig. 7(D)), with evident sub-cellular detail in the guard cells obtained compared to using the 8X configuration. Though the 3D printed lenses and their commercial counterparts are comparable in resolution, higher blurring is observed through the printed lenses as a result of aberrations and scattering. Similarly, for the cyanobacteria sample (Fig. 7(G)) the commercial and 3D printed lens 50X objectives are comparably close in resolution and contrast, though blurring is more prominent in the 3D printed case due to the previously mentioned aberrations and scattering. When adding a second 3D printed lens element, meaning 3D printed versions of lenses #3 and #4 have replaced their commercial counterparts, into the custom multi-element objective (Fig. 7(E) and (H)), a reduction in resolution is visible, specifically looking at the stomata complex in the *Hosta* sample. For the cyanobacteria filament the individual compartments are still clearly visible but the overall clarity of intracellular details is as expected slightly reduced.

## 4. Discussion

As shown in Fig. 5(B), faint ‘phantom’ images of the USAF target are observed across the 3D printed lens images, for example visible to the left edge of Fig. 5(B) where faint replicas of group 4 elements 2-6 can be seen. This scattering issue has been experimentally determined to be a diffraction effect, likely due to prominent voxel patterning obvious within the uncoated 3D printed lens itself. This generated effective diffraction grating throughout the bulk optic shows that though coating mitigates the staircase effect, the bulk print still influences image quality. The grating pattern exhibited in the uncoated 3D prints may be unique to the used family of printer, as uncoated 3D prints from higher resolution resin printers do not show observable voxel patterns resembling diffraction gratings. The phantom image effects are less obvious in dense samples, in particular Fig. 5(C) and Fig. 5(D), which show more blurred edges likely as a direct product of diffraction though with less discernible laterally displaced ‘phantom’ images. The brightfield images produced of stomata within the *Hosta*, cyanobacteria filament morphology, and onion cell structures using the 8X 3D printed objective are showing similar details to their commercial counterpart albeit with reduced contrast and resolution. For epi-fluorescence applications shown in Fig. 7, the 8X commercial PSF seen in Fig. 7(A) shows the expected radial symmetry, while the 3D printed version has scattering and inhomogeneity effects due to the aberrations induced through a spin-coated 3D printed lens and the volume scattering discussed earlier, as well as scattering due to any residual surface defects caused by inhomogeneities in the coated surface.

When moving to the ∼50X 4-lens objective configuration, it can be seen from Fig. 6 that an individual 3D printed lens #4 at this magnification achieves comparable contrast and resolution to the commercial equivalent, as the finest area of the USAF target are resolved in both cases as well as sub-cellular structural details, for example chloroplasts within the *Hosta* stomata guard cell walls. This is to be expected when considering the quality obtained from the 8X configuration, with aberration effects comparatively concurrent between the two different configurations. Figure 6 also exemplifies that two 3D printed imaging lenses, lenses #3 and #4, compound the aberrations which decreases the overall contrast and resolution, though details at micrometer scale are still distinguishable as shown by the USAF target in Fig. 6(C). However, Fig. 6 G exemplifies that this combination of 3D printed lenses makes identifying individual chloroplasts more challenging in comparison to both the commercial alternative and the single 3D printed lens example in Fig. 6(D). When evaluating the ∼50X epi-fluorescence imaging performance, the PSFs in Fig. 7(B) show similar results to the 8X objective, where the commercial PSF is radially symmetric as expected compared to the subsequent two 3D printed variants which both have significant aberrations. Next to this the PSFs of the 3D printed variants also show significant scattering around the central PSF point, likely originating from the volume grating effect seen in the printed lenses. Additionally, the ∼50X configuration required higher power than the 8X in order to obtain a similar signal to noise ratio, despite being a higher numerical aperture, which can be in part due to the increased number of optical elements within the 4-lens setup.

When comparing 3D printed optical element performance for both brightfield and fluorescence imaging, we observe similar artefacts and aberrations in both the USAF target images in brightfield, and the *Hosta* stomata cell images in fluorescence illumination conditions. So far, 3D printed optics literature has shown resolution target imaging and beam shaping results with comparable resolutions and uniformity to the imaging shown in this work. However, shown for the first time is the application of these manufacturing methods to biological specimen imaging with sub-cellular resolution, drawing attention to the potential for blood smear imaging and therefor low-cost diagnoses of blood diseases and field diagnostics. When comparing USAF target results using our 3D printed lens approach to other published research, our technique is comparable to other documented spin-coating post-processing approaches regarding resolution and contrast [15]. However, comparing our result to other optical additive manufacturing processing approaches, we can see that lens molding may offer superior resolution to spin-coating [15]. The low-cost manufacturing method provides ample opportunity for lens combinations as well as custom optics geometries and prescriptions. However, with each 3D printed lens added into the collection arm of the microscope, the contrast and resolution of the imaging system depletes as shown in brightfield (Fig. 6) and fluorescence imaging (Fig. 7).

## 5. Conclusions

We have shown that using low-cost manufacturing methods for 3D printed optical elements used as single and 4-lens microscope objectives allows collection of both brightfield and fluorescence information in transmission and epi-fluorescence illumination configurations, highlighting their promising potential for custom biological imaging applications. The surface profiles of 3D printed optics closely match their commercial equivalents with curvature radii meeting >90% conformity. While using a single 3D printed lens element in a microscope objective has shown comparable resolution and contrast to commercial glass lenses, we have also shown the impact of adding multiple 3D printed lenses into a custom microscope objective, leading to compounding aberrations in both brightfield and fluorescence imaging. Despite these additional aberrations, we have shown the fluorescence imaging performance using a multi-element 3D printed objective that allowed the resolving of inter-cellular compartments in *Hosta* as well as in cyanobacteria filament samples. The achieved sub-cellular resolution using 3D printed optics within the shown biological samples opens a new gateway into low-cost biological imaging applications in healthcare and field diagnostics.

## Data Availability

Data underlying the results presented in this paper are available in Ref. [27]

## Supplemental document

See Supplement 1 for supporting content.
